# Erosive Tooth Wear in Subjects with Normal Occlusion: A Pioneering Longitudinal Study up to the Age of 60

**DOI:** 10.3390/jcm12196318

**Published:** 2023-09-30

**Authors:** Henrique Campos Eto, Felicia Miranda, Daniela Rios, Heitor Marques Honório, Guilherme Janson, Camila Massaro, Daniela Garib

**Affiliations:** 1 Department of Pediatric Dentistry, Orthodontics and Public Health, Bauru Dental School, University of São Paulo, Bauru 17012-901, SP, Brazil; 2 Department of Orthodontics, Hospital for Rehabilitation of Craniofacial Anomalies, University of São Paulo, Bauru 17012-900, SP, Brazil

**Keywords:** tooth wear, dental erosion, permanent dentition, longitudinal studies, disease progression, age factors

## Abstract

The aim of this study was to assess the erosive tooth wear (ETW) at early and mature adulthood in subjects with natural normal occlusion. The sample consisted of 23 untreated subjects with normal occlusion. Dental models were taken longitudinally from the same subjects at 13 (T0), 17 (T1) and 60.9 years of age (T2) at a university. Evaluation of ETW was performed using a modified Basic Erosive Wear Examination (BEWE) index. Interphase changes were evaluated using Friedman and Dunn’s test. Ordinal logistic regression was used to assess the influence of sex, dental arch, tooth and dental surfaces on the erosive tooth wear. Linear regression was used to evaluate whether the ETW degree at T1 could discriminate the degree of ETW at T2. The significance level adopted was 5%. ETW showed a significant increase with aging. The median ETW index at T0, T1 and T2 was 2, 4 and 7, respectively. ETW was greater in males in the incisors and canines and on the incisal/occlusal and lingual tooth surfaces. No significant differences were found between the maxillary and mandibular arches. Subjects with severe ETW at mature adulthood had greater tooth wear at age 17. In conclusion, ETW significantly increased during aging in subjects with normal occlusion. The greater the degree of tooth wear at early adulthood, the greater the tooth wear at mature adulthood. Preventive care should be recommended during early adulthood in patients demonstrating erosive tooth wear in order to avoid worsening with aging.

## 1. Introduction

Throughout life, teeth are exposed to a broad spectrum of physical and chemical injuries, which contributes to a wide range of tooth wear [1]. Chemical factors can be extrinsic or intrinsic as the consumption of acidic foods or presence of stomach acids from reflux and regurgitation, respectively [2]. Parafunctional habits such as bruxism, regular mastication (attrition) and toothbrushing (abrasion) are mechanical or physical factors [3]. Erosive tooth wear (ETW) corresponds to a chemical–mechanical process of loss of tooth crown volume [1]. ETW starts when the enamel surface is softened by an acid, leaving a more liable surface to mechanical impacts, which partially remove the softened enamel layer [4].

Interactions of chemical/biological, behavioral, socioeconomic and educational factors were previously reported for tooth wear development [4]. Other local factors, such as multiple tooth loss, parafunctional habits and changes in mandibular movement patterns, can also influence the process [5]. ETW is a global problem demonstrating an increasing prevalence over time [6]. It is not yet clear whether there is a natural cumulative process that progresses throughout life or the speed rate of the wear changes depending on the impact of risk factors [7]. In other words, the differentiation between a physiological wear process due to aging from pathological ETW is very difficult [4]. This difficulty results from the impossibility of quantifying dental wear clinically [8,9] and the lack of clinical studies with long-term follow-up. Tooth wear has been detected and scored by several indexes. The Basic Erosive Wear Examination (BEWE) index was proposed by Bartlett and represents a simple and accurate tool for assessing tooth wear, guiding treatment decisions [8]. From a clinical point of view, the index should be repeated once a year in patients presenting risk factors.

The knowledge of erosive tooth wear (ETW) during aging is essential to identify whether the presence of wear in adolescence can predict a higher level of wear in mature adulthood. Assessing ETW longitudinally from adolescence to mature adulthood also contributes to identifying parameters of pathological wear. Most previous studies on ETW have been cross-sectional or short-term longitudinal in untreated malocclusions or a longitudinal study of tooth wear in orthodontically treated patients [6,10,11,12,13,14,15]. There is a literature gap in understanding the longitudinal changes in erosive wear during aging in subjects with acceptable and untreated occlusions. This understanding is very important to define the prognosis of ETW with aging and to plan preventive approaches.

Therefore, this study aimed to evaluate ETW over five decades, from adolescence to 60 years of age, in subjects with normal occlusion. Another objective was to assess if mature adults with severe tooth wear have earlier indicators of tooth wear in early adulthood. The null hypothesis was that the erosive tooth wear index is similar in adolescence, early and mature adulthood.

## 2. Materials and Methods

This retrospective study was approved by the Ethics Committee on Human Research of CAAE: 22082019.4.0000.5417 and written consent was obtained from all subjects. The study protocol was not registered.

The sample of this study was selected among a sample of 82 white subjects (39 male, 43 female) with normal occlusion recruited from 1967 to 1974 at the Orthodontic Department of Bauru Dental School, University of São Paulo. The inclusion criteria at T0 were as follows: clinical normal occlusion, complete permanent dentition, molar and canine Class I relationship, no crossbites, normal overjet and overbite, maximum 2 mm of incisor crowding, well-balanced faces, and no previous history of orthodontic treatment. First, the dental models were obtained at 13 years of age (T0). In the second recruitment stage, the same patients had dental models taken at 17 years of age (T1). The same subjects were recalled from 2015 to 2016 and dental models were obtained at 60 years of age (T2) [16]. From the initial sample of 82 subjects, thirty-eight patients were reached, eleven patients did not want to participate (8) or have lost all their teeth (3). Thirty-six patients were not found and eight had died. Twenty-nine subjects were enrolled during the recall at T2. Subjects with a history of orthodontic treatment were excluded. The final sample comprised 23 subjects (10 female, 13 male). The mean age at T0 was 13.06 (SD = 0.98), 17.56 at T1 (SD = 0.96) and 60.9 years at T2 (SD = 1.49). The mean follow-up period from T0 to T2 was 47.84 years.

Clinical evaluation was performed prior to dental impressions. The mean number of patients with tooth loss at 60 years of age was 2.1 teeth per subject. In general, posterior teeth were the most frequently absent compared to anterior teeth. Tooth loss was more frequent in the mandible compared to the maxilla. In addition, for a total of 42 missing teeth, only 11 were rehabilitated with implants/prosthesis. (Table 1) In the complete sample, 168 incisors, 87 canines, 136 premolars and 36 molars were assessed for erosive tooth wear.

All dental models were digitized using a R700 3-dimensional scanner (3Shape, Copenhagen, Denmark). Using the Ortho Analyzer 3-dimensional software (3Shape, Copenhagen, Denmark), digital dental models were analyzed for erosive tooth wear using the Basic Erosive Wear Examination (BEWE) index [8] and a modified version of the BEWE index.

**Figure 1:**
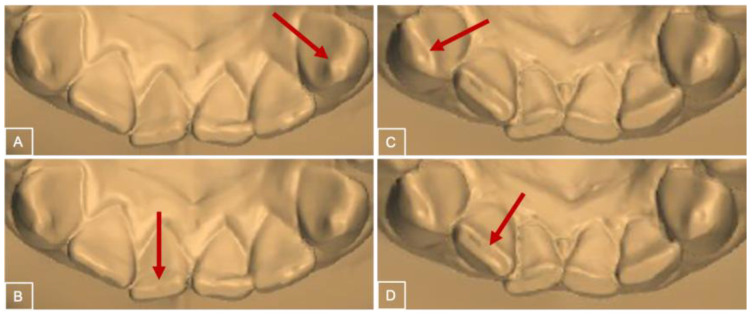
BEWE index. Score 1 (**A**), Score 2 (**B**), Score 3 (**C**) and Score 4 (**D**). The arrows present the location of the score.

As demonstrated in Figure 1, BEWE scores vary from no surface loss (score 0), initial loss of enamel surface texture (score 1), distinct defect with dentine loss in less than 50% of the surface area (score 2) or dentine hard tissue loss with more than 50% of the surface area (score 3) [8]. All teeth were examined, except the second and third molars, implants, and restored surfaces. The incisal/occlusal, buccal, and lingual surfaces were analyzed. The most severely affected surface in each sextant was recorded. The final score for each subject was calculated by the sum of the scores of the six sextants. The range of the score sum varied from 0 to 18.

A modification in the BEWE index was also applied to identify the most affected tooth regions and surfaces. All teeth and surfaces were analyzed and recorded individually. Teeth were grouped by regions: incisors (central and lateral incisors), canines, premolars (first and second premolars) and molars (first molars). Incisal/occlusal, buccal, and lingual surfaces were grouped. For the tooth region evaluation, the most severely affected surface of each tooth was considered.

The assessment was conducted by two trained examiners (H.C.E., F.M.) in two time points. Examiner calibration was performed using 35 original photographs of teeth with ETW with different degrees of severity. An experienced professional in the diagnosis and management of ETW (D.R.H.) together with the examiners went over the BEWE assessment. The percentage of diagnostic agreement between the examiners and the gold standard exceeded 95%. The calibration was conducted by a repeated examination after a 30-day interval and all photographs were reevaluated by the two examiners to assess intra and interexaminer agreement (κ > 0.85).

### Statistical Analyses

For the error study, 20% of the sample was randomly re-measured by both raters after a 21-day interval. Intra and interexaminer reproducibility were evaluated using the kappa coefficient.

Normal distribution was evaluated using the Shapiro–Wilk test. For the tooth wear score, normal distribution was not found. Nonparametric statistics were selected. Tooth wear scores were described using median and interquartile range (IQR) values considering the tooth surface and tooth region. The difference between the three time points was analyzed using Friedman test and Dunn’s Method for multiple comparisons.

An ordinal logistic regression analysis was performed at T2 considering tooth wear as a dependent variable and sex, maxillary/mandibular dental arch, tooth region and surfaces as independent variables. A linear regression analysis was performed considering tooth wear at T2 as a dependent variable and tooth wear at T1 as independent variables.

All the analyses were performed using SigmaPlot 12.0 (Systat Software Inc., San Jose, CA, USA), Statistica 10.0 (StatSoft Inc., Tulsa, OK, USA) and Jamovi 1.2 (Computer Software Inc., Sydney, Australia). The level of significance was set at 5%.

## 3. Results

Intra and interexaminer agreements were 0.90 and 0.91, respectively, presenting almost perfect agreements.

To evaluate erosive tooth wear throughout aging, the median over the three time points was used. The median BEWE score of the complete dentition in T0, T1 and T2 was 2, 4 and 7, respectively (Table 2). There was a significant increase in erosive tooth wear between phases with significant differences between all of them.

The ordinal logistic regression analyses are presented in Table 3. The analysis of ordinal logistic regression predicted the odds ratio (OR) of each variable to predict the tooth wear. Sex, dental arch, tooth region and surface were the predictors, whereas the tooth wear was the dependent variable. According to the results, sex was a predictor of erosive tooth wear (*p* = 0.003 *) (Table 3). Males had more ETW than females. Males were 1.54 times more likely than females to develop ETW (odds ratio = 1.54). In a comparison between the maxillary and the mandibular arch, a significant difference was found (Table 3).

The tooth region had an influence on erosive tooth wear at mature adulthood (T2). Incisors and canines had, respectively, 4.72- and 2.26-times higher chances to demonstrate ETW compared to premolars (Table 3). The premolars were used as a reference in the analysis for presenting the least ETW among the tooth regions. Evaluating the tooth crown surfaces, the incisal/occlusal and the lingual aspects had greater ETW compared to the labial/buccal surfaces at mature adulthood. The labial/buccal surface was used as a reference in this analysis. The incisal/occlusal and lingual surfaces had, respectively, 144.83- and 2.19-times greater chances (odds ratio) of demonstrating ETW compared to the buccal surface (Table 3).

Linear regression analysis reveals that the degree of ETW at T1 was a predictive factor for the degree of ETW at T2 (*p* < 0.001 *). On the other hand, the degree of ETW at adolescence (T0) could not predict the degree of ETW at T2. For this reason, at T2, subjects were divided into two groups demonstrating erosive tooth wear below and above the median of sample ETW (Table 4). As shown in Table 4, subjects with greater erosive tooth wear at mature adulthood (T2) already demonstrated greater erosive tooth wear at early adulthood (T1) (*p* < 0.018 *) (Table 4).

## 4. Discussion

Tooth wear indices remain the most convenient and reproducible method to grade wear severity. In the present study, a reproducible qualitative index was used to assess ETW [8,13]. The BEWE index is a partial scoring system recording the most severely affected surface in each sextant. Compared to other classical indexes, the BEWE system showed adequate reliability to score the severity of ETW [17,18]. Additionally, previous studies have shown good accuracy of the BEWE index [19,20]. Assessment of the BEWE index can be performed by direct oral examination, using dental photographs, conventional or digital dental models [13,21,22]. In our study using the digital dental model, the intra and interexaminer reproducibility of the BEWE index was adequate. These results are in accordance with previous studies [13,21]. One limitation of the BEWE index is that the scores do not differentiate wear at the level of enamel or dentine because the surfaces are scored independently of the exposed structure. In addition, the original BEWE index does not identify the tooth or surface that is compromised by ETW. To overcome this last limitation, a modification of the BEWE index was conducted in this study, in which all surfaces of all teeth were classified according to BEWE scores to allow identification of the most affected tooth region and surface.

Tooth wear is a common and universal outcome during the aging process [23]. No previous longitudinal study followed subjects with untreated normal occlusion until the seventh decade of life in order to evaluate erosive tooth wear. A significant increase in the BEWE score was observed from 13 to 17 and from 17 to 60 years of age (Table 2). The total BEWE index increased 5 points during 47 years of follow up. These results corroborate previous studies showing an increase in the BEWE score with increasing age [6,11,14]. A previous cross-sectional study assessed tooth wear from 20 to 70 years of age and found an increase in ETW from 3% to 17% [10]. In mature ages, severe tooth wear involving large dentin exposure was observed [24]. A previous study used a finite element analysis to determine the stress on molars and identify the mechanism of enamel damage in the grooves of the teeth during computer-simulated mastication [25]. During stimulated mastication, significant tensile stress was found to be concentrated on the mandibular molar enamel around the central groove and the foramen cecum [25].

The present data showed that men demonstrated greater levels of ETW compared to women (Table 3). This result is in accordance with previous cross-sectional and longitudinal studies [6,26,27,28]. The possible explanation is related to greater muscular strength and biting forces in men [27]. In addition, the sexual difference in tooth wear can also be related to differences in food preferences and type of behavior/lifestyle [29]. Compared to women, men are more likely to prefer sour foods and beverages with lower pH levels [29]. Very intense physical activities might cause changes in saliva composition and decreases in salivary flow rate [30]. Conversely, in other studies, the prevalence of ETW did not vary according to sex [11,31], or there was a higher prevalence of ETW in women, probably because the sample was younger than the sample of the present study [32].

**Figure 2:**
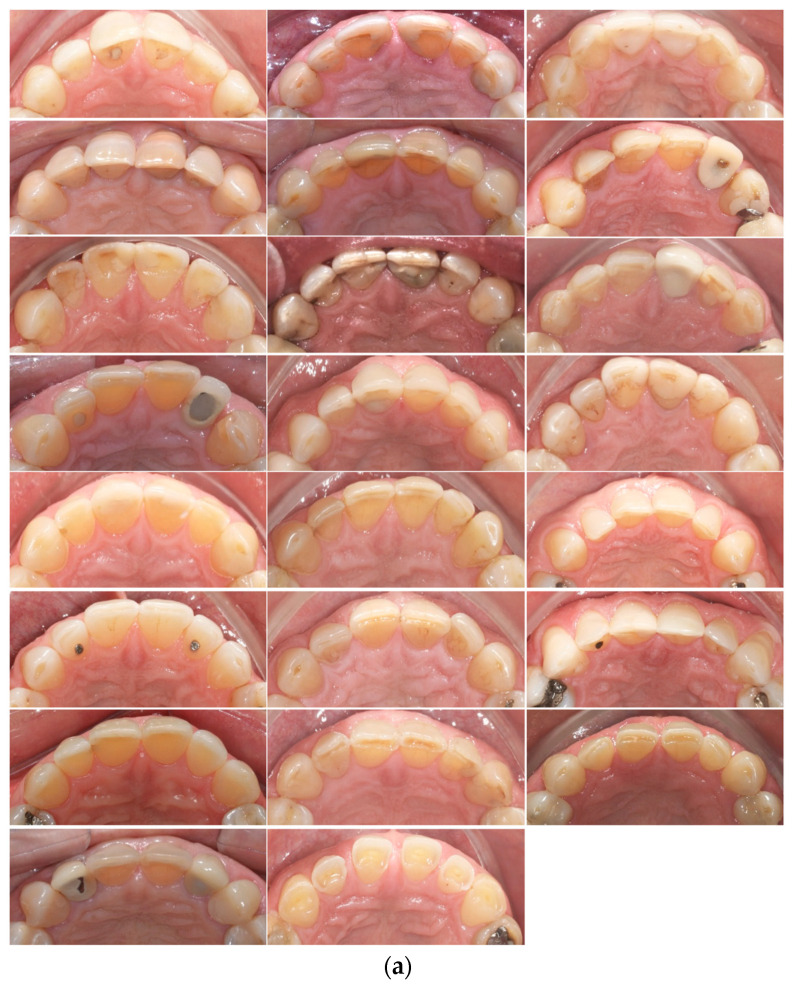

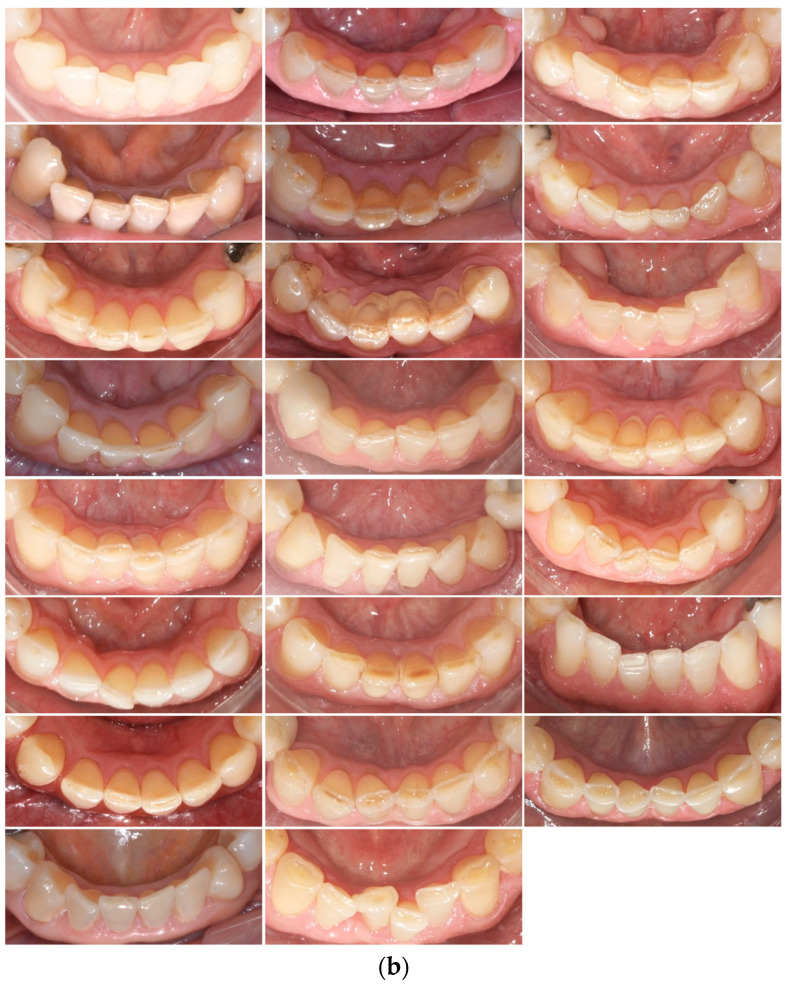
(**a**) Maxillary anterior teeth in all sample subjects at T2. (**b**) Mandibular anterior teeth in all sample subjects at T2. Figure order corresponds to the same sequence of patients demonstrated in (**a**).

Both dental arches were equally affected by tooth wear, possibly due to the masticatory reciprocity (Table 3). These results are corroborated by a previous study [33]. The most affected teeth in our study were the incisors and canines (shown in Figure 2a,b). Molar and premolars showed a similar level of tooth wear (Table 3). Previous studies also reported a predominance of tooth wear in the anterior teeth [11] [26,31,32,34]. In contrast, some authors have also found more ETW in the posterior teeth [12,35]. There are two possible explanations for the increased erosive tooth wear in the incisors and canines. The first assumption is that function and parafunction require more of the anterior than the posterior teeth [34,36]. During functional occlusion, the anterior and lateral excursions of the mandible are usually guided by the incisors and canines, respectively. Secondly, incisors and canines also have a restricted incisal surface for occlusal force and attrition distribution.

When tooth surfaces were analyzed, the incisal/occlusal areas were the most affected by ETW (Table 3). There is no consensus in the literature regarding the most affected surface. Previous studies corroborate our findings [27,28,32,33]. The incisal surfaces were probably the most affected due to the small area and functional and parafunctional mandible excursions [8,32]. The greater wear on the occlusal surface of molars was explained by both the contact with the antagonist teeth during mastication and the thinner enamel thickness on the occlusal surface [8,33]. On the other hand, a previous study demonstrated greater wear on the palate of the incisors due to episodes of vomiting and gastrointestinal reflux [37,38]. Other studies have reported relevant wear on the buccal related to erosive drinks and food [39].

Nystrom et al. reported that tooth wear of the primary anterior teeth at 5 years of age demonstrated low predictive values for tooth wear at 18 years of age, whereas wear at 14 years of age had a predictive value [40]. Subjects with greater tooth wear at early adulthood demonstrated more tooth wear at mature adulthood (Table 4). In view of these outcomes, when a young adult is identified with ETW, an investigation should be performed to identify risk factors that might be unbalancing the natural occurrence of wear. A detailed anamnesis evaluating the history of diseases, use of medications, drink and diet habits and presence of parafunctions should be accomplished.

The limitations of this study were the difficulties related to collecting longitudinal data [41,42], due to the extensive follow-up period of 50 years. In addition, at mature adulthood (T2), some patients exhibited many tooth losses and prosthesis had to be excluded. Another limitation is the small sample size that could have limited the logistic regression model analysis. Therefore, the results should be considered with caution. Erosive tooth wear is a current problem in modern society. There is an increased concern regarding the assessment of oral health impact on different dimensions of quality of life during the aging process [43]. Identification of the predictive risks for ETW in early adulthood is an important tool for the clinicians to prevent severe consequences in the dentition of patients that present with aging. Future longitudinal studies should assess the erosive tooth wear associated with functional occlusion, diet and lifestyle.

## 5. Conclusions

An increase in erosive tooth wear occurred with aging. From the second to the seventh decade of life, males, incisors/canines, and the incisal/occlusal and lingual surfaces were more affected by erosive tooth wear. No differences were observed between the maxillary and mandibular dental arches. Preventive care for ETW should be recommended at early adulthood in patients demonstrating erosive tooth wear in order to avoid worsening with aging.

## Figures and Tables

**Table 1:** Tooth losses in mature adulthood (T2).

	Teeth	Lost and Not Replaced	Dental Implant	Prosthesis	Evaluated	Total
Maxillary	16/26	10	1	7	22	46
	15/25	3	1	8	34	
	14/24	1	0	8	37	
	13/23	0	0	1	45	
	12/22	0	1	3	42	
	11/21	0	0	3	43	
Mandibular	36/46	6	11	11	18	46
	35/45	1	5	7	33	
	34/44	2	2	3	39	
	33/43	0	0	2	44	
	32/42	0	0	0	46	
	31/41	0	0	0	46	

**Table 2:** Interphase changes for the BEWE score (Friedman test and Dunn’s method).

	T0–13 Years			T1–17 Years			T2–61 Years			*p* Value
**BEWE score**	Median	25%	75%	Median	25%	75%	Median	25%	75%	<0.001 *
	2.00 ^A^	1.00	2.00	4.00 ^B^	3.00	5.00	7.00 ^C^	5.00	10.00	

Different letters represent statistically significant differences. * Statistically significant at *p* < 0.05.

**Table 3:** Influence of sex, dental arch, tooth region and dental surface on the erosive tooth wear at T2 (ordinal logistic regression analysis).

Predictor		Estimate	SE	Z	*p* Value	Odds Ratio	95% CI	
							Lower	Upper
Sex (male versus female)		0.43	0.14	3.02	0.003 *	1.54	1.16	2.03
Dental arch (maxillary versus mandibular)		0.24	0.14	1.72	0.086	1.27	0.97	1.66
Tooth Region	Incisor-premolar	1.51	0.19	8.13	<0.001 *	4.72	3.26	6.90
	Canine-premolar	0.82	0.21	3.80	<0.001 *	2.26	1.49	3.45
	Molar-premolar	0.58	0.34	1.71	0.087	1.78	0.90	3.39
Surface	Incisal/occlusal-buccal	4.98	0.24	20.94	<0.001 *	144.83	91.91	233.45
	Lingual-buccal	0.78	0.18	4.33	<0.001 *	2.19	1.54	3.13

* Statistically significant at *p* < 0.05.

**Table 4:** Comparisons of erosive tooth wear at early adulthood (T1) between subgroups with a ETW below and above the median at mature adulthood (T2) (*t*-tests).

Subgroups	N	T1-ETW Mean	SD	*p* Value	95% CI	
					Lower	Upper
BEWE score below T2 median	13	3.308	1.182	0.018 *	2.59	4.02
BEWE score above T2 median	10	4.700	1.418		3.69	5.71

* Statistically significant at *p* < 0.05; SD = Standard Deviation.

## Data Availability

The data analyzed during the current study are available from the corresponding author on a reasonable request.

## References

[1] Schlueter N., Amaechi B.T., Bartlett D., Buzalaf M.A.R., Carvalho T.S., Ganss C., Hara A.T., Huysmans M.-C.D., Lussi A., Moazzez R. (2020). Terminology of erosive tooth Wear: Consensus report of a workshop organized by the ORCA and the Cariology research group of the IADR. Caries Res..

[2] Moazzez R., Anggiansah A., Bartlett D.W. (2005). The Association of Acidic Reflux above the Upper Oesophageal Sphincter with Palatal Tooth Wear. Caries Res..

[3] Thordarson A., Zachrisson B.U., Mjor I.A. (1991). Remodeling of canines to the shape of lateral incisors by grinding: A long-term clinical and radiographic evaluation. Am. J. Orthod. Dentofac. Orthop. Off. Publ. Am. Assoc. Orthod. Its Const. Soc. Am. Board Orthod..

[4] Lussi A., Carvalho T.S. (2014). Erosive tooth wear: A multifactorial condition of growing concern and increasing knowledge. Erosive Tooth Wear.

[5] d’Incau E., Couture C., Maureille B. (2012). Human tooth wear in the past and the present: Tribological mechanisms, scoring systems, dental and skeletal compensations. Arch. Oral Biol..

[6] Holbrook W.P., Arnadottir I.B., Hloethversson S.O., Arnarsdottir E., Jonsson S.H., Saemundsson S.R. (2014). The Basic Erosive Wear Examination (BEWE) applied retrospectively to two studies. Clin. Oral Investig..

[7] Bartlett D. (2016). A personal perspective and update on erosive tooth wear—10 years on: Part 1—Diagnosis and prevention. Br. Dent. J..

[8] Bartlett D., Ganss C., Lussi A. (2008). Basic Erosive Wear Examination (BEWE): A new scoring system for scientific and clinical needs. Clin. Oral Investig..

[9] Ganss C., Young A., Lussi A. (2011). Tooth wear and erosion: Methodological issues in epidemiological and public health research and the future research agenda. Community Dent. Health.

[10] Van’t Spijker A., Rodriguez J.M., Kreulen C.M., Bronkhorst E.M., Bartlett D.W., Creugers N.H. (2009). Prevalence of tooth wear in adults. Int. J. Prosthodont..

[11] Vered Y., Lussi A., Zini A., Gleitman J., Sgan-Cohen H.D. (2014). Dental erosive wear assessment among adolescents and adults utilizing the basic erosive wear examination (BEWE) scoring system. Clin. Oral Investig..

[12] Bartlett D.W., Lussi A., West N.X., Bouchard P., Sanz M., Bourgeois D. (2013). Prevalence of tooth wear on buccal and lingual surfaces and possible risk factors in young European adults. J. Dent..

[13] Marro F., De Lat L., Martens L., Jacquet W., Bottenberg P. (2018). Monitoring the progression of erosive tooth wear (ETW) using BEWE index in casts and their 3D images: A retrospective longitudinal study. J. Dent..

[14] Ganss C., Klimek J., Giese K. (2001). Dental erosion in children and adolescents—A cross-sectional and longitudinal investigation using study models. Community Dent. Oral Epidemiol..

[15] Knight D.J., Leroux B.G., Zhu C., Almond J., Ramsay D.S. (1997). A longitudinal study of tooth wear in orthodontically treated patients. Am. J. Orthod. Dentofac. Orthop..

[16] Massaro C., Miranda F., Janson G., de Almeida R.R., Pinzan A., Martins D.R., Garib D. (2018). Maturational changes of the normal occlusion: A 40-year follow-up. Am. J. Orthod. Dentofac. Orthop. Off. Publ. Am. Assoc. Orthod. Its Const. Soc. Am. Board Orthod..

[17] Mulic A., Tveit A.B., Wang N.J., Hove L.H., Espelid I., Skaare A.B. (2010). Reliability of two clinical scoring systems for dental erosive wear. Caries Res..

[18] Margaritis V., Mamai-Homata E., Koletsi-Kounari H., Polychronopoulou A. (2011). Evaluation of three different scoring systems for dental erosion: A comparative study in adolescents. J. Dent..

[19] Olley R.C., Wilson R., Bartlett D., Moazzez R. (2014). Validation of the Basic Erosive Wear Examination. Caries Res..

[20] Bartlett D., Dattani S., Mills I., Pitts N., Rattan R., Rochford D., Wilson N.H.F., Mehta S., O’Toole S. (2019). Monitoring erosive toothwear: BEWE, a simple tool to protect patients and the profession. Br. Dent. J..

[21] Alaraudanjoki V., Saarela H., Pesonen R., Laitala M.L., Kiviahde H., Tjaderhane L., Lussi A., Pesonen P., Anttonen V. (2017). Is a Basic Erosive Wear Examination (BEWE) reliable for recording erosive tooth wear on 3D models?. J. Dent..

[22] Wohlrab T., Flechtenmacher S., Krisam J., Saure D., Wolff D., Frese C. (2019). Diagnostic Value of the Basic Erosive Wear Examination for the Assessment of Dental Erosion on Patients, Dental Photographs, and Dental Casts. Oper. Dent..

[23] Carvalho T., Lussi A. (2017). Age-related morphological, histological and functional changes in teeth. J. Oral Rehabil..

[24] Bartlett D., O’Toole S. (2019). Tooth wear and aging. Aust. Dent. J..

[25] Dejak B., Bołtacz-Rzepkowska E. (2023). Mechanism of enamel damage in the grooves of molars during mastication. Dent. Med. Probl..

[26] Dugmore C.R., Rock W.P. (2004). The prevalence of tooth erosion in 12-year-old children. Br. Dent. J..

[27] El Aidi H., Bronkhorst E.M., Truin G.J. (2008). A longitudinal study of tooth erosion in adolescents. J. Dent. Res..

[28] McGuire J., Szabo A., Jackson S., Bradley T.G., Okunseri C. (2009). Erosive tooth wear among children in the United States: Relationship to race/ethnicity and obesity. Int. J. Paediatr. Dent..

[29] Gambon D.L., Brand H.S., Veerman E.C. (2012). Dental erosion in the 21st century: What is happening to nutritional habits and lifestyle in our society?. Br. Dent. J..

[30] Mulic A., Tveit A.B., Songe D., Sivertsen H., Skaare A.B. (2012). Dental erosive wear and salivary flow rate in physically active young adults. BMC Oral Health.

[31] Gurgel C.V., Rios D., Buzalaf M.A., da Silva S.M., Araujo J.J., Pauletto A.R., de Andrade Moreira Machado M.A. (2011). Dental erosion in a group of 12- and 16-year-old Brazilian schoolchildren. Pediatr. Dent..

[32] Wang P., Lin H.C., Chen J.H., Liang H.Y. (2010). The prevalence of dental erosion and associated risk factors in 12-13-year-old school children in Southern China. BMC Public Health.

[33] Sun K., Wang W., Wang X., Shi X., Si Y., Zheng S. (2017). Tooth wear: A cross-sectional investigation of the prevalence and risk factors in Beijing, China. BDJ Open.

[34] Schierz O., Dommel S., Hirsch C., Reissmann D.R. (2014). Occlusal tooth wear in the general population of Germany: Effects of age, sex, and location of teeth. J. Prosthet. Dent..

[35] Ab Halim N., Esa R., Chew H.P. (2018). General and erosive tooth wear of 16-year-old adolescents in Kuantan, Malaysia: Prevalence and association with dental caries. BMC Oral Health.

[36] Liu B., Zhang M., Chen Y., Yao Y. (2014). Tooth wear in aging people: An investigation of the prevalence and the influential factors of incisal/occlusal tooth wear in northwest China. BMC Oral Health.

[37] Auad S.M., Waterhouse P.J., Nunn J.H., Steen N., Moynihan P.J. (2007). Dental erosion amongst 13-and 14-year-old Brazilian schoolchildren. Int. Dent. J..

[38] Mangueira D.F., Sampaio F.C., Oliveira A.F. (2009). Association between socioeconomic factors and dental erosion in Brazilian schoolchildren. J. Public Health Dent..

[39] Lussi A., Schaffner M., Hotz P., Suter P. (1991). Dental erosion in a population of Swiss adults. Community Dent. Oral Epidemiol..

[40] Nystrom M., Kononen M., Alaluusua S., Evalahti M., Vartiovaara J. (1990). Development of horizontal tooth wear in maxillary anterior teeth from five to 18 years of age. J. Dent. Res..

[41] Bishara S.E., Treder J.E., Damon P., Olsen M. (1996). Changes in the dental arches and dentition between 25 and 45 years of age. Angle Orthod..

[42] Harris E.F. (1997). A longitudinal study of arch size and form in untreated adults. Am. J. Orthod. Dentofac. Orthop..

[43] Skośkiewicz-Malinowska K., Noack B., Kaderali L., Malicka B., Lorenz K., Walczak K., Weber M.-T., Mendak-Ziółko M., Hoffmann T., Ziętek M. (2016). Oral health and quality of life in old age: A cross-sectional pilot project in Germany and Poland. Adv. Clin. Exp. Med..

